# Use of Mental Health Apps by Patients With Breast Cancer in the United States: Pilot Pre-Post Study

**DOI:** 10.2196/16476

**Published:** 2020-04-15

**Authors:** Philip I Chow, Shayna L Showalter, Matthew Gerber, Erin M Kennedy, David Brenin, David C Mohr, Emily G Lattie, Alisha Gupta, Gabrielle Ocker, Wendy F Cohn

**Affiliations:** 1 Department of Psychiatry and Neurobehavioral Sciences University of Virginia Charlottesville, VA United States; 2 Department of Surgery University of Virginia Charlottesville, VA United States; 3 Department of Engineering Systems and Environment University of Virginia Charlottesville, VA United States; 4 Department of Public Health Sciences University of Virginia Charlottesville, VA United States; 5 Center for Behavioral Intervention Technologies Northwestern University Chicago, IL United States

**Keywords:** breast cancer, mental health, mHealth

## Abstract

**Background:**

Nearly half of the patients with breast cancer experience clinically significant mental distress within the first year of receiving their cancer diagnosis. There is an urgent need to identify scalable and cost-efficient ways of delivering empirically supported mental health interventions to patients with breast cancer.

**Objective:**

The aim of this study was to evaluate the feasibility of in-clinic recruitment for a mobile phone app study and to evaluate the usability and preliminary impact of a suite of mental health apps (IntelliCare) with phone coaching on psychosocial distress symptoms in patients recently diagnosed with breast cancer.

**Methods:**

This pilot study adopted a within-subject, 7-week pre-post study design. A total of 40 patients with breast cancer were recruited at a US National Cancer Institute–designated clinical cancer center. Self-reported distress (Patient Health Questionnaire-4) and mood symptoms (Patient-Reported Outcomes Measurement Information System depression and anxiety scales) were assessed at baseline and postintervention. App usability was assessed at postintervention.

**Results:**

The minimum recruitment threshold was met. There was a significant decrease in general distress symptoms, as well as symptoms of depression and anxiety, from baseline to postintervention. Overall, participants reported high levels of ease of app use and learning. Scores for app usefulness and satisfaction were reinforced by some qualitative feedback suggesting that tailoring the apps more for patients with breast cancer could enhance engagement.

**Conclusions:**

There is a dire need for scalable, supportive interventions in cancer. The results from this study inform how scalable mobile phone–delivered programs with additional phone support can be used to support patients with breast cancer.

**International Registered Report Identifier (IRRID):**

RR2-10.2196/11452

## Introduction

### Background

Nearly 50% of the women diagnosed with breast cancer report clinically significant levels of distress (ie, elevated symptoms of depression or anxiety) within the first year of receiving their cancer diagnosis [1-4]. Untreated symptoms of depression and anxiety in patients with breast cancer lead to poor quality of life [5], increased mortality [6,7], and high economic costs [8]. Although therapies that emphasize skills acquisition, such as cognitive behavioral therapy and acceptance-based therapy, have demonstrated efficacy in reducing distress in patients with breast cancer [9-12], almost half of the patients with breast cancer report unmet supportive care needs [13-16]. One reason is the reliance on in-person delivery of mental health services, which poses numerous barriers, such as high financial cost [17], high time investment [18,19], social stigma [20], and a severe shortage of trained therapists [21-23]. Despite increased efforts by clinicians and researchers to assess for distress during the cancer treatment process, distress management through mobile technology remains an overlooked component of care [14,24,25].

### Mobile Phone Apps for Patients With Cancer

Mobile phone apps are frequently cited as a potential method of extending effective care in a cost-effective manner [26-28]. Given that 81% of American adults own a mobile phone [29], it is an ideal platform from which to deliver brief, empirically supported interventions to anyone who needs them. Models of internet interventions [30] and behavioral intervention technologies [31] highlight key strengths of mobile health (mHealth) interventions: portability, accessibility, and the ability to program an automated intervention to adapt to a user’s input. Numerous randomized controlled trials demonstrate the efficacy of app-based interventions in reducing the symptoms of depression and anxiety [32-36], including those that are coupled with support from a coordinator [32,37,38]. However, empirical reviews of apps for patients with cancer [25,39] fail to identify any publicly available mental health intervention that target patients with breast cancer. Thus, despite the potential scalability and impact of an app-based intervention that teaches distress management skills to patients with breast cancer, more work is needed.

### App Design and Coaching to Promote Engagement

An app-based mental health intervention can be deployed where and when a patient needs it most, guiding users through brief and practical skills training to manage their distress. However, there are some weaknesses to app interventions, including software bugs, ownership of a compatible mobile device, and poor engagement and usage. Specifically, many apps suffer from poor engagement for a variety of reasons, such as requiring lengthy engagement times that do not match user preferences [40]. In reality, people use apps in short, frequent bursts and tend to prefer apps that support a limited set of tasks [41]. Thus, an app intervention that is designed to provide quick and targeted interventions can potentially fit well with patients with breast cancer who are receiving active cancer treatment and who must deal with the inevitable sequelae of anticancer care, including time constraints and conflicts with work and outside activities [35].

Studies suggest that pairing an app with human support (eg, coaching via phone, SMS text messaging) can further increase engagement and usage, thereby promoting outcomes [37,38]. On the basis of the Efficiency Model of Support [38], a human coach can support participants in using and benefiting from an app intervention. Coaches work with users to set goals and target potential points at which users may fail to benefit from the app (ie, addressing obstacles to effective use), which increases accountability and promotes engagement [38].

The aim of this study was to conduct a pilot study that evaluated a set of brief, targeted app interventions that promote mental health. The IntelliCare platform is a collection of apps that utilize an elemental, skills-based approach to improving mental health [32,42]. Table 1 contains descriptions of the IntelliCare apps and their purposes. Many of the exercises contained in the apps can be completed in less than a minute. Exercises are meant to be intuitive, requiring few instructions to complete, and most of these exercises can be found on the first screen that is presented by the app. Each app has a *Help* feature that contains educational and technical content regarding the specific app in question. A total of two trials of 8-week interventions showed significant and substantial reductions in depression and anxiety symptom severity among noncancer patients with average app use of 195 to 216 times, with a median use of less than 1 min [32,42]. However, these results may not be generalizable to patients with breast cancer who face unique challenges and life circumstances, which makes them potentially unique from other populations.

There were two broad aims of this study. The first was to examine, in a single-group pre-post design, the feasibility and usability of the IntelliCare apps in patients recently diagnosed with breast cancer to inform a larger trial. We examined recruitment and retention rates to inform a potential future randomized trial. On the basis of the considerations of the size of the clinic from which participants were recruited, as well as the decision to recruit patients early in the breast cancer diagnostic pathway (at a time when it may not be appropriate to participate in a study that requires an immediate face-to-face consent and app download process), a threshold of 1 to 2 participants per week was the threshold to determine feasibility of in-clinic recruitment for a larger study [43]. The second aim was to examine the usability and preliminary impact of the IntelliCare apps in reducing distress in patients recently diagnosed with breast cancer. Note, this study initially sought to recruit the caregivers of patients with breast cancer; however, because of low enrollment, we decided to exclude the caregivers from the analyses. It was hypothesized that patients newly diagnosed with breast cancer would have decreases in general distress symptoms, as well as depression and anxiety symptoms, over a 7-week intervention period [43]. Quantitative and open-ended feedback was collected at the end of the study to evaluate usability and satisfaction of using the apps and coaching.

**Table 1:** Description of IntelliCare apps, their objectives, and which apps were available for each type of mobile phone platform at the time of the study.

App name	Objective	Mobile phone platform
Aspire	Promotes awareness of and striving toward personal goals and values. Helps users identify their values and keep track of their progress.	Android
Day to Day	Promotes knowledge about ways to bolster mood. Users receive a daily stream of knowledge tidbits and are prompted to build on a theme every day (eg, cultivate gratitude and problem solve).	Android and iOS
Daily Feats	Promotes goal setting and attainment. An in-app calendar allows users to track their successes and identify new tasks to complete.	Android and iOS
Worry Knot	Promotes knowledge about worry and provides an interactive exercise to decrease worry. The app also tracks the user’s progress and provides tailored feedback on ways to distract oneself from worrying thoughts.	Android and iOS
Social Force	Encourages users to identify supportive individuals in their life. The app prompts users to reach out to these people for encouragement.	Android
My Mantra	Increases self-efficacy and a positive perspective of oneself. The app prompts users to come up with personal mantras and to construct personalized photo albums that serve as reminders of these mantras.	Android and iOS
Thought Challenger	Increases the ability to identify and challenge negative thinking patterns. Guides users through a cognitive restructuring exercise and tracks the output of past exercises.	Android and iOS
iCope	Promotes coping and positive reinforcement by having users write and send themselves messages when encouragement is most needed.	Android
Purple Chill	Increases relaxation skills by providing a library of mindfulness and guided meditation audio files.	Android
MoveMe	Promotes mood through physical activity. The app prompts users to schedule exercises and provides instructional videos and lessons to increase motivation to exercise.	Android
Slumber Time	Promotes healthy sleeping by prompting users to keep an active sleep diary. The app also provides a checklist of things to do before bedtime to promote healthy sleep habits.	Android
Boost Me	Promotes positive mood by having users schedule positive activities throughout the day. A mood tracker allows users to see their progress and the impact of different activities on their mood.	Android

## Methods

### Overview

This was a single-group, 7-week pre-post study of patients with breast cancer in the United States. The decision to use a 7-week duration was based on the duration of brief face-to-face psychotherapy (typically 6-8 weeks) and previous reviews finding that the duration of app interventions usually range between 6 days and 8 weeks [44]. All participants received the IntelliCare apps and coaching. Self-report measures were obtained at baseline and postintervention to examine mental health outcomes. Additional measures were administered at the end of the study to evaluate user satisfaction and ways to improve the intervention for a future trial.

### Participants

A total of 40 female patients with breast cancer (age: mean 56.8 years, SD 11.6 years) actively receiving cancer treatment were enrolled over a course of 29 weeks. Among those that indicated their race, most participants self-identified as white (31/38, 82%), followed by black (4/38, 11%), Hispanic (1/38, 3%), American Indian or Alaska Native (1/38, 3%), and multiracial (1/38, 3%). The median number of days from cancer diagnosis to study enrollment was 21 days. Among those who reported their breast cancer stage, 11% (3/28) of the patients were diagnosed with stage 0, 25% (7/28) of the patients were diagnosed with stage 1, 39% (11/28) of the patients were diagnosed with stage 2, and 25% (7/28) of the patients were diagnosed with stage 3. Rural-urban commuting area (RUCA) codes V3.0 from the United States Department of Agriculture were determined using participant zip codes. RUCA codes range from 1 (most metropolitan) to 10 (most rural) and are based on US Census tract data of population density, urbanization, and daily commuting. In this study, 47% (17/36) of the participants resided in an area characterized as most urban or metropolitan (RUCA=1), 42% (15/36) of the participants resided in an area characterized as metropolitan or micropolitan (RUCA=2-6), and 11% (4/36) of the participants resided in an area characterized as small town or rural (RUCA=7-10).

To limit barriers to entry, inclusion criteria were limited to the following: (1) patient diagnosed with breast cancer within the last 2 months, (2) age at least 18 years, (3) proficient in English at a sixth-grade level, and (4) has a mobile phone or is willing to carry one around if provided. Participants were not required to have a minimum level of familiarity with mobile devices or technology. Note, a total of 12 caregivers were also enrolled and were provided the same apps. Owing to the low number of caregivers enrolled, in this study, we focused on data obtained from patients with breast cancer.

### Procedure

**Figure 1:**
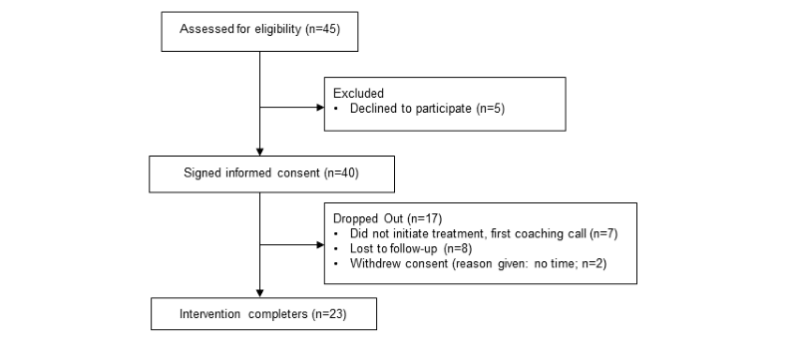
Study flow.

Patients with breast cancer were recruited from a breast care clinic in a US National Cancer Institute–designated clinical cancer center. Surgical oncologists and nurses handed out a study flyer to patients with breast cancer during a normal scheduled visit. Patients had an opportunity to speak to a research staff member, who provided more details about the study and answered questions. If an eligible patient expressed interest in participating, the patient was led through the consenting process by a research staff member. Research staff described the aims of the study, introduced the IntelliCare apps, and reviewed the study timeline. After providing written consent, participants scheduled a 30-min coaching call (see description below) that took place sometime within the next 10 days with a research staff member, which marked the initiation of their treatment in the study. Participants were guided to download the apps in the consent session, but they were told not to open them until the coaching call. Participants then completed a battery of measures that took approximately 10 to 15 min to complete. Following the initial coaching call, participants received an SMS text message (via Qualtrics’ SMS tool) every week to remind them to try two new exercise modules in the app. After 7 weeks (postintervention), participants completed another battery of self-report measures on the Web. They also provided feedback about their experiences of using the app and coaching. See Figure 1 for information on patient recruitment and flow. Participants were compensated with a US $50 gift card for providing feedback. Informed consent was obtained from all individual participants included in the study. All procedures performed were in accordance with the ethical standards of the University of Virginia Institutional Review Board (IRB-HSR# 20648) and with the 1964 Helsinki declaration.

### Materials

Participants used their own personal mobile phone. A concerted effort was made to include both Android and iOS users into the study, given the differences between users of these platforms in previous work [26]. A total of 3 participants did not own a mobile phone or have an appropriate mobile phone plan that enables downloading and using a native mobile phone app, and they were provided with a Samsung S7 Android phone with an unlimited data plan. These individuals were able to use the phones for nonstudy purposes. All IntelliCare apps were available for Android users, and a total of five apps (ie, Thought Challenger, Worry Knot, Daily Feats, My Mantra, and Day To Day) were available to iOS users at the time of the study. Android users were instructed to try two new apps every week for the first 6 weeks and to use any combination of apps for the seventh week. iOS users were instructed to try one new app every week for the first 5 weeks and to use any combination of apps for the sixth and seventh weeks. Among the initially enrolled 40 female patients with breast cancer, 31 had an Android phone and 9 had an iOS phone.

### Phone Coaching

A manualized coaching protocol was adopted from a previous IntelliCare study [32], based on the Efficiency Model of Support [38]. The goals of coaching are to address usability issues, increase engagement with the app, promote fit of the intervention by assessing the needs of patients with cancer, promote knowledge of the skills found in the app, and encourage implementation of the skills in daily life [38]. Usability concerns include issues related to the usability of the intervention, fit of the intervention tool to one’s needs, knowledge of how to use the intervention, and implementation failures. Coaches were instructed to focus on app-related issues and to refrain from engaging in traditional counseling with participants. An initial coaching call (designed to last 30 min) focused on orienting participants to downloading and using the app, setting expectations of the coach’s role, assessing how the apps may meet participants’ needs, and building rapport. Participants were told that they could contact coaches at any time with any app-related questions. A total of 2 coaches with a bachelor’s degree were trained and closely monitored by the lead author (PC). Finally, an unstructured 10-min phone call 3 weeks after the initial coaching call served as a check-in to make sure that participants did not have any lingering concerns or questions.

### Measures

#### General Psychological Distress

The Patient Health Questionnaire-4 (PHQ-4) [31] is widely used in cancer settings as a brief screener of general distress and symptom burden, and it is well validated in both general and clinical samples [31,32]. Individuals are asked to rate (0=not at all and 3=nearly every day) the degree to which they experienced different states (eg, “Little interest or please in doing things”) over the past 2 weeks. Scores range from 0 to 12; a score of 6 to 8 indicates moderate mood symptoms, whereas a score of 9 and higher indicates severe mood symptoms. The PHQ-4 was administered at baseline and postintervention.

#### Symptoms of Depression and Anxiety

Depression symptoms were assessed with the 4-item scale from the Patient-Reported Outcomes Measurement Information System (PROMIS) [30] 29-item profile version 2.0 (PROMIS-29 Profile v2.0). PROMIS, a US National Institutes of Health Roadmap program, provides sensitive and reliable measures of patient-reported outcomes. Participants are asked to report (1=never and 5=always) the degree to which they experienced various depressed states (eg, “I felt worthless” and “I felt hopeless”) over the past 7 days. Continuous anxiety symptoms were assessed with the 4-item scale from the PROMIS-29 Profile v2.0. Participants are asked to report (1=never and 5=always) the degree to which they have experienced different anxious states (eg, “My worries overwhelmed me” and “I felt fearful”) over the past 7 days. The PROMIS scales were administered at baseline and postintervention. Consistent with PROMIS scoring recommendations, raw summed scores were converted into T-scores for analyses, with higher scores indicating greater symptom levels.

#### User Feedback

User feedback was assessed at postintervention. The USE-short form [45] was used to examine usability and satisfaction of the IntelliCare app suite as a whole. It is composed of 21 items that assess user experience (eg, “I would recommend it to a friend,” “It is easy to learn to use it,” and “It is simply to use”), which comprises the domains of usefulness, ease of use, ease of learning, and satisfaction. Items are scored on a 7-point Likert scale (1=strongly disagree and 7=strongly agree). The USE measure is a well-validated scale that is commonly used to evaluate the user experience of mHealth interventions [46,47].

Participants also provided open-ended feedback during telephone interviews with the research staff. The interviews covered the following topics related to the apps: general impressions, design quality, technical needs, and design suggestions to promote app implementation and usage. In addition, participants were asked to provide feedback on the following aspects of phone coaching: general experience with coaches, usefulness of coaching, additional or unmet coaching needs, suggestions to improve the coaching experience.

### Data Analysis

Outcome data were stored in a secured Qualtrics server for highly sensitive data. Analyses were done in SPSS Statistics for Windows, version 25.0 (IBM Corp, Armonk, NY).

Quantitative user data were analyzed descriptively by obtaining means and SDs. Qualitative feedback data were reviewed for emerging themes. Specifically, responses were coded on the domains of the following: (1) ways to improve the design and user interface of the apps, (2) the specific apps that were most helpful (and why), (3) the specific apps that were least helpful (and why), (4) obstacles and barriers to using the apps, and (5) ways to improve the usefulness of coaching calls [43].

Paired *t* tests were used to analyze self-reported outcome data among patients with breast cancer [43] and to examine whether the use of the IntelliCare apps was associated with changes in distress and symptoms of depression and anxiety before vs after the 7-week intervention.

## Results

### Feasibility of In-Clinic Recruitment

See Figure 1 for information on study flow. A total of 45 patients with breast cancer were assessed for eligibility, of which 40 signed the informed consent form. A total of 23 patients with breast cancer completed the 7-week intervention, and 17 individuals prematurely dropped out because of noninitiation of treatment (ie, failure to complete the first coaching call), lost contact, and withdrawal of consent because of the perceived time burden of being in the study.

Patients with breast cancer were recruited over a span of 29 weeks, from March 2018 to September 2018. Thus, the minimum recruitment threshold of in-clinic recruitment of 1 to 2 participants was met (note, the recruitment rate is higher if the 12 caregivers who provided informed consent are included in the total count). Incremental adjustments were made during the trial to increase the efficiency of the patient recruiting process. Specifically, we were able to identify key personnel (ie, nurses and patient navigators) and clinic procedures to more easily identify eligible patients. These changes did not have an impact on the study procedures after the informed consent form was signed. A paper discussing the challenges and potential solutions of in-clinic recruitment for mHealth pilot studies, based on our experience of conducting this study, is forthcoming.

### Distress and Mood Symptoms

Table 2 contains the descriptive statistics of psychosocial outcomes. On the basis of the PROMIS T-scores, there were significant reductions in symptoms of depression (t_22_=2.35; *P*=.03; 95% CI 0.32 to 5.03; Cohen *d*=0.52) over the 7-week intervention period. Although there was also a reduction in symptoms of anxiety (t_22_=2.05; *P*=.05; 95% CI −0.05 to 7.52; Cohen *d*=0.45), this did not reach significance.

Consistent with the previous findings, patients with breast cancer reported significant reductions in general psychological distress (PHQ-4) [48] over the 7-week intervention period (t_22_=2.61; *P*=.02; 95% CI 0.23 to 2.03; Cohen *d*=0.55). At baseline, among those who completed the 7-week study, 22% (6/28) of patients reported at least a moderate level of distress, whereas 8% patients (3/38) reported at least a moderate level of distress at postintervention.

### App Usage

The median number of total IntelliCare app launches was 97, roughly equal to two app launches per day over the course of the trial. Table 3 contains additional app usage statistics for the individual apps.

**Table 2:** Means and SDs of psychosocial outcomes at each time point, along with the results of paired *t* tests.

Outcomes	Baseline, mean (SD)	Postintervention, mean (SD)	*P* value
Depression symptoms	53.77 (9.60)	51.09 (10.45)	.03
Anxiety symptoms	60.26 (8.84)	56.53 (9.67)	.05
General distress	3.96 (2.65)	2.83 (2.48)	.02

**Table 3:** Median number of app launches and median duration of app launches of individual IntelliCare apps.

App name	App launches (number)	Duration (in seconds)
Aspire	10.5	20
Day to Day	20	48
Daily Feats	33	38
Worry Knot	11.5	32.5
Social Force	2	27.5
My Mantra	5	21
Thought Challenger	7	19
iCope	7	27
Purple Chill	24	17
MoveMe	6.5	20
Slumber Time	9	35
Boost Me	10	78

### Feedback

Patients with breast cancer rated the apps highly in terms of ease of use (mean 5.62, SD 1.3) and ease of learning (mean 5.67, SD 1.6) on the USE-short form. In general, participants had favorable yet relatively lower ratings for the domains of usefulness (mean 4.26, SD 1.8) and satisfaction (mean 4.05, SD 1.9).

A closer examination of the qualitative feedback of the patients with breast cancer supported the quantitative findings. Thematic analyses revealed that many participants found the apps very easy to use. A common theme was that despite not being computer or technologically savvy, participants found the apps to be fairly easy to use. Participants also reported that they generally liked the simple, straightforward design, which helped them to navigate the apps. Another theme that emerged was the utility of phone coaching. Participants reported that their interactions with coaches were pleasant and helpful in using the apps. There was general agreement that coaches helped patients with breast cancer feel supported while in the study, and the frequency and duration of phone calls were not viewed as overly burdensome, although none wanted more phone calls with coaches. It is worth noting that the sentiment of phone coaching as useful was not unanimous, as a minority of participants felt that phone coaching was unnecessary.

Additional themes hinted at ways to improve the IntelliCare apps for patients with breast cancer. A common theme was that participants reported that the look and feel of the apps, including the content (eg, examples of distressing thoughts), were not relevant to someone with breast cancer (eg, “the apps are not relevant to someone going through cancer...some questions or things don’t pertain to cancer” and “you should tailor [the apps] to situational cancer”). Another recurring theme was related to the timing of app use in relation to cancer stage and treatment progress. Many patients with breast cancer reported that the apps may be most useful for patients diagnosed with a more severe stage of cancer (eg, stage 3 or 4) or those undergoing chemotherapy.

## Discussion

### Principal Findings

Overall, patients with breast cancer found the apps easy to use and navigate. Feedback obtained at the end of the study highlighted several areas for potential improvement, all of which entail making the apps more relevant for patients with breast cancer and their experiences.

This study established the feasibility of recruiting patients newly diagnosed with breast cancer to engage in an mHealth intervention from a relatively small breast surgery oncology clinic. Receiving a cancer diagnosis is a life-changing moment for many individuals. Psychosocial distress is known to peak around the time of breast cancer diagnosis and the early stages of cancer treatment [49,50]. Thus, recruiting individuals around the time of diagnosis is a significant challenge to evaluating mobile app interventions. To meet the minimum threshold of feasibility (1 to 2 participants per week) [43], our team needed to adjust to the structure and flow of the clinic. For example, a significant amount of time was devoted to introducing the study to nurses and patient navigators. Research assistants had to coordinate with the clinic staff to present the study to eligible patients. Researchers who are interested in conducting an mHealth pilot study in patients newly diagnosed with cancer are encouraged to factor in clinic space, staff, and patient flow when designing their study and calculating enrollment figures.

Patients with breast cancer identified several areas of improvement for a future trial. As the IntelliCare apps were designed for use in the general population, many patients reported wanting the appearance and content of the apps to reflect their experiences. A wealth of studies demonstrate the importance of tailoring digital interventions for end users [51,52]. In recent years, there has been a notable rise in the awareness of breast cancer through media and social campaigns [53,54], leading many patients with breast cancer to strongly identify with their diagnosis [55,56]. The most prominent theories of behavior change [57-59] stress that interventions that are perceived as personally relevant are most likely to succeed in changing people’s behavior. Thus, to increase engagement with an app-delivered intervention for patients with breast cancer, it is important to tailor it in ways that are meaningful to those end users. For example, adding examples of cancer-related worrying thoughts (eg, “My cancer will never go away” and “I’m not strong enough to go through chemotherapy”) to the Thought Challenger app may improve engagement with the app. Future work should also consider tailoring the app based on cancer stage and timing of treatment. For example, introducing the apps to patients right before starting chemotherapy may provide them with the needed coping skills during cancer treatment. Finally, although the patients with breast cancer generally found the coaching to be useful, none reported wanting more coaching calls, and a few participants found the coaching to be unnecessary. On the basis of this feedback, future studies may consider only providing coaching to a subset of patients with breast cancer who are in greatest need. For example, by leveraging a Sequential, Multiple Assignment, Randomized Trial [60], individuals who struggle to engage with the apps could be identified and provided with coaching. Providing support on an individual basis maximizes the scalability of app-based interventions by providing a more efficient use of resources.

Pilot studies are often conducted to obtain an effect size estimate to power a larger trial; therefore, this study’s findings should not be overinterpreted in light of the relatively small sample. However, the results suggest that the IntelliCare apps have a moderate effect (based on Cohen *d*) in reducing mood and anxiety symptoms in patients recently diagnosed with breast cancer. Although the effect sizes obtained in this study are smaller than those reported in previous IntelliCare trials among the general population [32,42], they are comparable with the effect sizes of other mental health interventions (eg, mindfulness and in-person therapy) that have been tested among patients with breast cancer [61,62]. Achieving even a modest reduction of mental health symptoms may justify the expanded use of digital mental health interventions in patients with breast cancer, given their scalability, cost, and accessibility.

### Limitations and Future Directions

The findings from this study should be interpreted in light of several limitations. Given the size and characteristics of the sample in this study, these findings may not be generalizable to other cancer populations (eg, pancreatic and lung). The findings related to the potential impact of IntelliCare on distress symptoms should be replicated in a larger sample of patients with breast cancer. As this was a single-arm trial, we cannot rule out the possibility that the observed improvements were because of factors other than IntelliCare, such as the natural course of the problems. It will be important to evaluate the IntelliCare apps in a randomized controlled trial among patients with breast cancer. Thus, it is important to not overinterpret the study’s findings because of the absence of a control condition. As this study was conducted in a US National Cancer Institute–designated clinical cancer center, the findings regarding in-clinic recruitment feasibility have limited generalizability to other settings that may not possess as many resources. In addition, although the participants in this study were guided to download the apps during the informed consent session, future work should examine the benefits of providing more structure in teaching users how to navigate treatment apps. Finally, as there were more apps available to Android users than iOS users, it is hard to determine which apps were most efficacious in reducing distress symptoms. Future work should consider standardizing the order in which apps are tried, to allow for a better understanding of the effect of each app on psychosocial outcomes.

Finally, although the dropout rate of individuals in this study was generally at par with other app interventions, it was noticeably higher than that reported in previous IntelliCare studies in the general population. This may be attributed to the fact that individuals in this study were dealing with the stress of a recent breast cancer diagnosis. Similarly, the app usage rates were considerably lower than those reported in previous IntelliCare trials. This is consistent with research indicating that intervention impact and engagement generally decrease when moving from general to clinical samples [63]. Future studies should continue to explore the ways to address dropout in populations at high risk of dropout, such as providing added human support or connecting patients with additional resources in their community. Despite a higher dropout rate and a decrease in app usage in this study than those reported in previous IntelliCare trials in the general population, findings suggest that patients with breast cancer are still able to use, and benefit from, an app-delivered mental health program.

### Conclusions

Mobile phone apps hold significant promise to overcome barriers in providing psychosocial care for patients with breast cancer [64-67]. However, relatively few publicly available apps have been empirically validated for treating mood symptoms [33,44], and those that have been validated have not been tested among patients with breast cancer [25]. IntelliCare, which has been rigorously studied in the general population [32,68,69], has the potential to make a significant public health impact by providing support to a large population of patients with breast cancer.

## Abbreviations

mHealth: mobile health
PHQ-4: Patient Health Questionnaire-4
PROMIS: Patient-Reported Outcomes Measurement Information System
RUCA: rural-urban commuting area
